# “Yellow-dragon Wonderful-seed Formula” for hyperuricemia in gout patients with dampness-heat pouring downward pattern: a pilot randomized controlled trial

**DOI:** 10.1186/s13063-018-2917-8

**Published:** 2018-10-11

**Authors:** Xiao Ning Yu, Hai Yan Wu, Yuan Ping Deng, Guang Tong Zhuang, Bang Huan Tan, Yan Zhou Huang, Shi Yun Tang, Xiang Tu, James B Jordan, Sen Zhong

**Affiliations:** 1 Basic Medical College of Chengdu University of Traditional Chinese Medicine, Chengdu, 611137 Sichuan Province China; 2 Department of Gerontology, Sichuan Academy of Medical Sciences & Sichuan Provincial People’s Hospital, Chengdu, 610072 Sichuan Province China; 3 Department of Internal Medicine, Traditional Chinese Medicine Hospital of Fushun County, Fushun, 643200 Sichuan Province China; 4 Department of Internal Medicine, Traditional Chinese Medicine Hospital of Pidu District, Chengdu, 611730 Sichuan Province China; 5 Department of Internal Medicine, Traditional Chinese Medicine Hospital of Kaizhou District, Chongqing City, 405400 China; 6 College of Clinical Medicine, Chengdu University of Traditional Chinese Medicine, Chengdu, 610075 Sichuan Province China; 7 National Traditional Chinese Medicine Clinical Research Base for Diabetes Mellitus/Teaching Hospital of Chengdu University of Traditional Chinese Medicine, Chengdu, 610072 Sichuan Province China; 8 Administration Committee of Affiliated Hospitals of Chengdu University of Traditional Chinese Medicine, Chengdu, 611137 Sichuan Province China

**Keywords:** *Yellow-dragon Wonderful-seed* formula, Chinese herbal medicine formula, Gout, Hyperuricemia, Randomized controlled trial, Gypsum Fibrosum

## Abstract

**Background:**

In Traditional Chinese Medicine (TCM) theories, the typical clinical manifestations of gout are attributed to the “dampness-heat pouring downward.” Therefore, TCM practitioners always consider prescribing the formulae which are believed to clear heat and drain dampness for the management of gout. This clinical trial aims: (1) to determine the hypouricemic effect of “Yellow-dragon Wonderful-seed Formula” (YWF) decoction in gout patients with dampness-heat pouring downward pattern and (2) to determine if gypsum could provide additional significant benefits to YWF.

**Methods:**

A total of 72 hyperuricemic individuals with gout and dampness-heat pouring downward pattern were included with 62 of them completing the trial. Participants were randomly assigned to the YWF group, the YWF + gypsum group, or the allopurinol group. YWF and YWF + gypsum decoctions were orally administered for four weeks. Allopurinol was also orally administered for four weeks as the active control. Serum uric acid (sUA) level was the primary outcome measure. Urine urate level, scores on the SF-36 scale, erythrocyte sedimentation rate (ESR), X ray film, and C reactive protein (CRP) level were the secondary outcome measures.

**Results:**

Compared with the values at week 0, YWF and YWF + gypsum did not significantly decrease the sUA level at each weekend reading. YWF, YWF + gypsum, and allopurinol decreased the urine urate levels and there were significant differences between the YWF group and the YWF + gypsum group. All the changes in the eight structures of SF-36 during the intervention period were not significantly different among the three groups and there was no significant difference in the CRP level among the three groups at each weekend reading.

**Conclusions:**

YWM, which modified on the basis of *Two Wonderful Herbs Powder* (*2WHP*), does not show significant hypouricemic effect. There is a possibility that Gypsum Fibrosum may provide additional effects to YWF in decreasing the urine urate levels but cannot add benefits to YWF in other outcome measures.

**Trial registration:**

Chinese Clinical Trial Registry, ChiCTR-TRC-12001933. Registered on 10 February 2012.

**Electronic supplementary material:**

The online version of this article (10.1186/s13063-018-2917-8) contains supplementary material, which is available to authorized users.

## Background

Epidemiological evidence from New Zealand, the USA, the UK, and China suggests that gout is becoming more prevalent [1]. The incidence of gout is approximately 1–2% in western countries, affecting > 3% of adults in the US [1, 2], which is very similar to that in China (1%) [3]. Gout is commonly characterized by hyperuricemia and acute arthritis, tophi, interstitial nephritis, and joint deformity resulting from deposition of sodium urate crystals.

At the beginning of the 21st century, according to the epidemiology of the eastern city of Nanjing (Jiangsu province), the mean concentration of serum uric acid (sUA) was 342.3 ± 87.3 μmol/L in Chinese men and 252.2 ± 77.8 μmol/L in Chinese women [4]. The incidence of hyperuricemia was 17.6% in men and 9.3% in women; gout occurred in 1.98% and 0.72%, respectively [4].

An investigation carried out in the northern province of Hebei in 2004 reported that the incidence of hyperuricemia was 15.8% in 1215 male individuals [5]. An article published in 2012 reported that the incidence of hyperuricemia was 24.73% in 1929 men and 10.79% in 686 women in Guizhou province, located in south China [6].

In Traditional Chinese Medicine (TCM) theories, the typical clinical manifestations of gout are attributed to the “dampness-heat pouring downward.” Both dampness and heat are the pathogenic factors and they have distinct characteristics: dampness is similar to water and heat is similar to fire. When heat invades gout patients, it results in a red, swollen joint. In the original Chinese medical text, the *Huangdi Neijing* says, “When dampness attacks the body, it first impairs the lower part of the body.” Dampness tends to move downward, like water. Therefore, the disease caused by dampness usually involves the lower part of the body. Dampness usually affects the lower extremities in gout patients, leading to attacks on the feet, ankle, or knees.

Therefore, TCM practitioners always consider prescribing the formulae which are believed to clear heat and drain dampness for the management of gout. Theoretically, dampness can be drained by diuretic TCM or dried by using TCM bitter in taste. Heat can be eliminated by TCM of cold property.

The typical diuretic TCM formula is *Two Wonderful Herbs Powder* (*2WHP*). In fact, there is a series of formulae developed from the *2WHP* – the *Three Wonderful Herbs Pill* (*3WHP*) is modified from the *2WHP* by adding one TCM (Cyathulae Radix) and the *Four Wonderful Herbs Pill* (*4WHP*) is also modified from the *2WHP* by adding two TCM (Cyathulae Radix and Coix seeds).

Current reports demonstrated that the series formulae modified from *2WHP* are effective for hyperuricemia in gout (see below).

*2WHP:*

The experiment in vivo by Kong et al. [7] demonstrated that the hypouricemic effect of *2WHP* was “equal to that of the reference drug allopurinol” in hyperuricemic mice pretreated with oxonate.

In the article by Li [8], 98 individuals with gout received modified *2WHP* for 14 days. The results showed that the levels of sUA decreased below normal values in 64 participants, accounting for 65.3%. Lv et al. [9] reported that the water extract of *2WHP* could inhibit the production of hepatic urate and promote the excretion of urine urate in hyperuricemic rats induced by oxonic acid potassium salt. The underlying mechanisms may be related to the downregulation of the messenger RNA (mRNA) and protein levels of hepatic xanthine oxidase (XOD) and renal mouse urate transporter 1 (mURAT1) regulated by *2WHP*.

*3WHP:*

When expanding *2WHP* to *3WHP* by adding Cyathulae Radix, some case reports showed positive results. Lu et al. [10] reported that modified *3WHP* was given to 30 gout patients for ten days and the sUA values of 25 patients decreased below normal limits and were maintained for at least two years. Shan et al. [11] carried out an in vivo study to explore the mechanisms of *3WHP* for inflammation of acute gouty arthritis. Their results showed that *3WHP* could reduce the inflammation in the ankle of rats with acute gouty arthritis and this was related to the inhibition of TNF-α, IL-6, and IL-8 levels in the ankle synovial tissues of gout rats which were associated with the downregulation of NF-ΚB P65 protein expression by *3WHP*.

*4WHP:*

Hu et al. [12] reported *4WHP* could enhance the renal urate excretion by effectively reversing oxonate-induced alterations in renal mURAT1, mouse glucose transporter 9 (mGLUT9), and organic anion transporter 1 (mOAT1) mRNA and protein levels. The conclusion was that their findings “suggest that *4WHP* processes uricosuric and nephroprotective actions by regulating renal organic ion transporters in hyperuricemic animals.”

In a randomized controlled trial (RCT), 178 participants were randomized to either the modified *4WHP* group or the western medicine group. The treatment period was 14 days. The sUA level of the *4WHP* group decreased from 585.93 ± 93.93 μmol/L to 319.13 ± 87.63 μmol/L. There was no significant difference between the *4WHP* group and western medicine group. Two individuals suffered from slight diarrhea in the *4WHP* group [13]. In another RCT, 120 participants were randomly assigned to either the modified *4WHP* group (*n* = 60) or to the allopurinol group (*n* = 60). The UA and CRP levels were determined after a one-month intervention period. The authors concluded that modified *4WHP* could “significantly improve the symptoms and signs of gouty arthritis and decrease the levels of UA and CRP. It is good for gouty arthritis” [14].

As mentioned above, modified *2WHP* appears to be an excellent approach to hyperuricemia in gout patients. Published literature also suggested that gypsum (Pinyin Name: Shi Gao; Latin Name: Gypsum Fibrosum) played an important role for hyperuricemia in gout patients [15–17]. Generally, current clinical evidence concerning TCM for gout is methodologically problematic [8, 10, 13, 14]. We decided to put the *2WHP* and other TCM together to create a new formula, i.e. *YWF*, and test its clinical efficacy with a RCT of desirable methodology.

## Methods

### Aims

We carried out a RCT to evaluate YWF for the treatment of hyperuricemia in gout patients with the specific TCM pattern, i.e. dampness-heat pouring downward pattern. This was also to determine whether Gypsum Fibrosum could provide significant additional benefits to YWF.

### Design and setting of the study

The present clinical study was a pilot RCT where there were three arms and the allocation ratio among the three groups was 1:1:1. This pilot trial was neither a superiority test nor a non-inferiority test, it was an exploratory trial. The pilot RCT was conducted at six centers in Sichuan Province and Chongqing City, China.

### Participants

Participants must have a physician diagnosis of gout [18] and hyperuricemia (sUA > 420 μmol/L) and be aged 18–70 years. If the patient had already received treatment for gout, he/she had to undergo a two-week washout period; only those whose sUA remained > 420 μmol/L after the washout period could be included. Only the patients differentiated as dampness-heat pouring downward pattern were included. The dampness-heat pouring downward pattern was confirmed by clinical symptoms and signs manifested as red, swollen, hot, and painful acute joint arthritis, red tongue, yellow and greasy tongue coating, and smooth pulse [19]. The pattern must be differentiated by at least two trained doctors of TCM. Patients were excluded if they had any of the following: pregnancy or lactation; allergic constitution, or an allergic history to test TCM or allopurinol; serum creatinine > 1.5 mg/dL; elevated values of ALT twice as high as the normal upper limit; severe deformity or stiffness of gouty arthropathy resulting in disability; arrhythmia of clinical significance; or a history of alcohol abuse. The patients were also excluded if they had severe cerebrovascular, kidney, liver, or hematopoietic system co-morbidities, cancer or mental disorders; taken concurrent hypouricemic medications, azathioprine, 6-mercaptopurine, medications containing aspirin (> 325 mg) or salicylate; or had participated in other clinical trials within the past three months.

### Interventions

The typical TCM formula to clear heat and dry dampness is *Three Kinds of Kernels Decoction* (*TKKD*) and cardamom is the monarch herb of *TKKD.* We therefore, added cardamom to *4WHP* to make the new formula become more appropriate for the dampness-heat pouring downward pattern. Because TCM experts believe that blood stasis plays an important role in gout, we added *Pheretima* to promote blood circulation and to dredge the collateral. The TCM ingredient of the new formula is shown in Table 1 and we call it “Yellow-dragon Wonderful-seed Formula” (YWF), a reference to *Phellodendron bark* in Chinese which is called “Yellow cypress” and *Pheretima* called “earth dragon.” As some TCM experts reported, gypsum plays a special role in the treatment of gout [16, 17]. We designed an independent arm with YWF + gypsum. The daily dose of gypsum was 15 g.

**Table 1:** Ingredients TCM of YWF

Pinyin name	Latin name	English name	Daily dose (g)
Di Long	Pheretima	Earthworm	10
Dou Kou	*Amomum kravanh Pirre ex Gagnep.*	Amomum, cardamon	6
Huang Bai	*Cortex Phellodendri Chinensis*	Cortex Phellodendri, Phellodendron bark	10
Cang Zhu	*Atractylodes Lancea (Thunb.) DC.*	Atractylodes, sword-like attractylodes rhizome, Chineseatractylodes rhizome	9
Yi Yi Ren	*Coix lacryma-jobi L.var.ma-yuen(Roman.)Stapf(Yi Yi)*	Coix seeds,Job’s tears	20
Chuan Niu Xi	*Cyathula officinalis Kuan.*	Cyathula, medicinal cyathula root	10

In this study, 72 individuals were randomly assigned to receive either YWF decoction, YWF + gypsum decoction, or allopurinol. All patients were advised not to consume purine-rich diets. TCM was purchased from Sichuan Rejuvenation Hall Pharmaceutical Co., Ltd., China. Professor HouLin Xia from the College Pharmacy, Chengdu University of TCM, confirmed that all TCM ingredients were the right herbal species recorded in the Chinese Pharmacopeia (2015 Edition) [20]. Suining (Sichuan, China) FDA assayed the quality standards of all ingredients TCM and provided quality reports which confirmed that their quality standards meet the Chinese Pharmacopeia (2015 Edition) requirements. All TCM ingredients were decocted together for 30 min at 120 °C with an automatic boiling and packaging machine, using three packages of decoction (100 mL/package). The TCM decoction was taken orally three times daily (100 mL each time) for four weeks. The starting dose of allopurinol was 100 mg day^−1^ in week 1 and increased to 200 mg during weeks 2–4.

### Outcomes

The level of sUA was the primary outcome, which was measured by enzymatic method with a Beckman (USA, AU5400) automatic biochemical analyzer every four weeks. The assay agent was purchased from ZhongSheng Corporation, Beijing, China. The secondary outcome measures included the urine urate measured every four weeks by the same analyzer and agent for sUA, scores on SF-36 which were measured at week 0 and week 4, the ESR was measured by the Westergren method every four weeks, the CRP was measured with a CRP automatic analyzer (QUIKREAD GO, Finland) and its matched agent kits every four weeks, and the X ray film examined at week 0 and week 4. We recorded acute flares during the treatment period. We designed serum concentration of xanthine and hypoxanthine as the additional outcome measures in the study protocol, but they were not assayed due to the insufficiency of funding.

### Sample size

As this study was a pilot trial, each arm of the RCT had 24 participants.

### Randomization

#### Sequence allocation

The randomization sequence was generated with an SAS software.

#### Allocation concealment

The randomization sequence was concealed and disseminated with opaque envelopes.

#### Implementation

The Good Clinical Practice (GCP) center of the Teaching Hospital of Chengdu University of TCM provided the randomization sequence. TCM doctors at six collaborating centers enrolled patients and the assistant nurses disseminated the randomization envelopes.

### Blinding

Blinding was not used due to the obvious difference between YWF decoction and allopurinol. However, the statisticians were blind to the study design.

### Statistical methods

Intention-to-treat (ITT) analysis was used to analyze data and the primary population for assessing efficacy was the full-analysis set (FAS). Patients who took at least one dose of the study medications and had at least one value on treatment were included by the FAS. The last-observation-carried-forward method was used to impute the missing data, whereby missing values were replaced by the last non-missing value. The baseline characteristics among the three groups were analyzed by one-way analysis of variance (ANOVA). Repeated measures and multivariate analysis of variance of the general linear model were used to determine the changes in sUA, urine urate, and CRP. The scores on SF-36 scale were analyzed by analysis of covariance. SPSS software (version 18.0, SPSS Inc., Chicago, IL, USA) was used to analyze statistics and *P* < 0.05 was considered significant.

## Results

### Participant flow

**Fig. 1:**
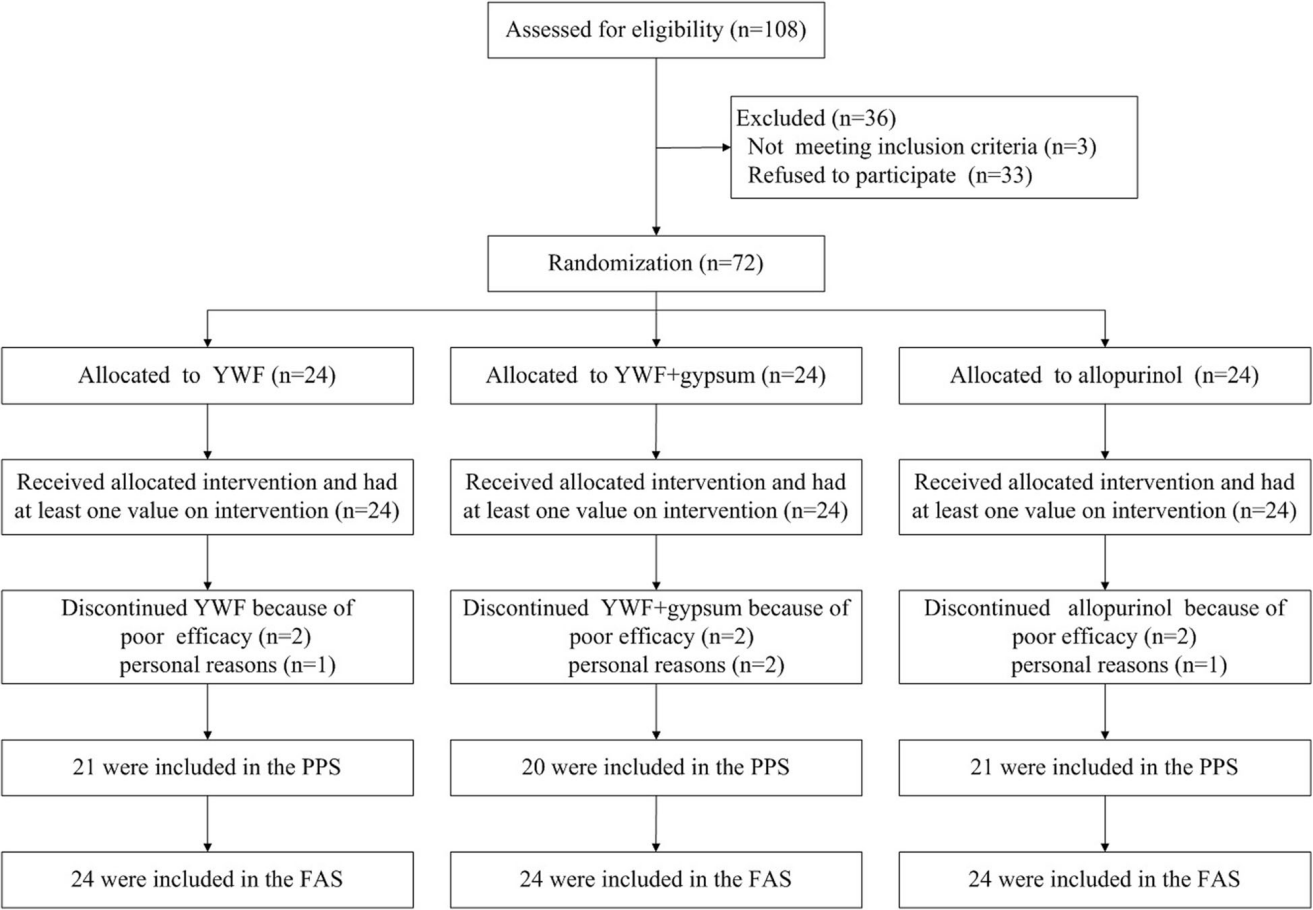
Participant flow. A total of 72 patients were randomized to the three arms. All 72 participants received the allocated intervention and therefore all 72 were included in the FAS. Ten individuals dropped out and therefore 62 participants were included in the PPS

A total of 72 male participants were involved in this study and ten dropped out during the study period (Fig. 1).

### Recruitment

The recruitment period was from 1 March 2012 to 15 December 2014.

### Baseline data

The baseline characteristics of the three groups of patients were comparable (*P* > 0.05) and shown in Table 2.

**Table 2:** The baseline characteristics of the FAS population of the three groups of patients

Characteristic		YWF	YWF + gypsum	Allopurinol	*F* value
Age (years)		45.33 ± 9.86	46.13 ± 10.75	49.21 ± 9.47	1.00
Gout history (months)		39.42 ± 29.00	55.25 ± 36.58	42.96 ± 32.38	1.54
sUA (μmol/L)		562.29 ± 108.29	585.46 ± 100.06	618.00 ± 114.27	1.62
Urine urate (mmol/24 h)		7.80 ± 0.37	7.79 ± 0.33	7.78 ± 0.31	0.01
CRP (mg/L)		13.13 ± 2.63	14.03 ± 3.40	13.15 ± 1.13	0.33
ESR (mm/h)		8.96 ± 4.99	8.79 ± 3.16	9.88 ± 6.96	0.29
SF-36 scale	BP	25.00 ± 14.52	31.71 ± 11.74	26.96 ± 9.54	1.95
	RP	27.08 ± 23.22	22.92 ± 19.39	25.00 ± 23.31	0.21
	PF	78.96 ± 7.07	81.04 ± 9.44	77.50 ± 10.11	0.95
	VT	73.13 ± 7.91	75.83 ± 9.85	75.42 ± 9.20	0.63
	SF	41.15 ± 22.87	43.23 ± 17.28	42.71 ± 20.16	0.07
	RE	19.43 ± 21.79	26.38 ± 24.04	24.99 ± 22.52	0.95
	GH	64.79 ± 13.95	66.04 ± 12.70	59.71 ± 14.43	1.44
	MH	43.67 ± 15.78	46.17 ± 15.78	46.42 ± 16.23	0.22

### Numbers analyzed

The per-protocol set (PPS) included 21 participants in the YWF arm, 20 in the YWF + gypsum arm, and 21 in the allopurinol arm. The FAS included 24 participants in each of the three arms.

### Outcomes

The data analysis on the FAS was largely consistent with the PPS.

#### sUA

**Fig. 2:**
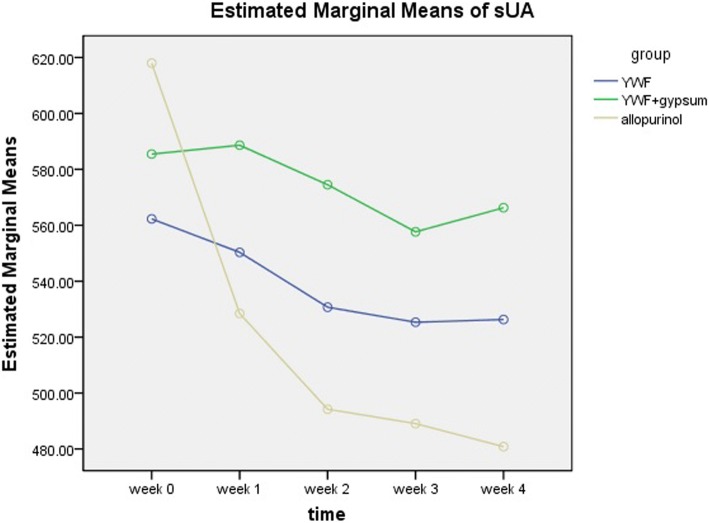
Changes in sUA of the three intervention groups. The sUA level of the YWF arm and YWF + gypsum arm did not significantly decrease at each reading, whereas allopurinol significantly reduced the sUA level. There was no significant change among the three arms at each reading

Compared with the values at week 0, YWF and YWF + gypsum did not significantly decrease the sUA levels at each weekend reading (Table 3, Fig. 2). There was no significant difference between YWF and YWF + gypsum at each reading (*P* > 0.05).

**Table 3:** Changes in sUA (mean ± SD, μmol/L)

		Week 0	Week 1	Week 2	Week 3	Week 4
PPS	*YWF* arm	547.38 ± 97.32	539.24 ± 121.62	522.43 ± 143.97	516.29 ± 155.89	517.38 ± 157.96
	YWF + gypsum arm	570.35 ± 93.61	572.90 ± 148.29	568.05 ± 140.46	548.75 ± 164.78	559.10 ± 202.94
	Allopurinol arm	618.76 ± 115.64	517.43 ± 124.03*	482.10 ± 144.27*	476.24 ± 136.72*	466.81 ± 141.27*
FAS	*YWF* arm	562.29 ± 108.30	550.33 ± 120.16	530.71 ± 143.63	525.33 ± 154.39	526.29 ± 156.15
	YWF + gypsum arm	585.46 ± 100.06	588.62 ± 144.79	574.50 ± 157.62	557.67 ± 176.13	566.29 ± 206.08
	Allopurinol arm	618.00 ± 114.27	528.46 ± 125.58*	494.21 ± 145.64*	489.08 ± 139.65*	480.83 ± 144.34*

*In comparison with week 0, there was a statistically significant difference (*P* < 0.05)

#### Urine urate

**Fig. 3:**
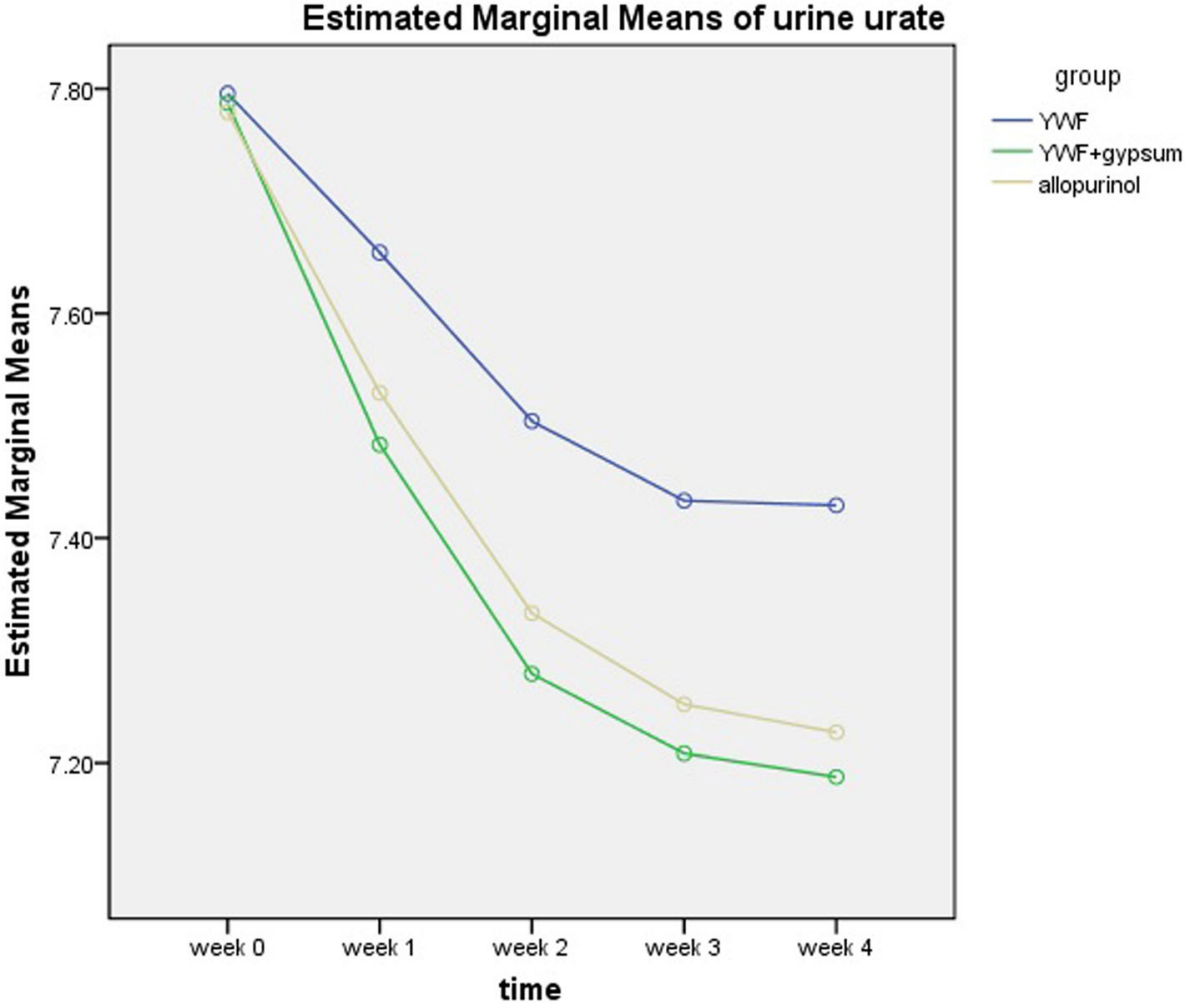
Changes in urine urate of the three intervention groups. The level of urine urate of all three arms significantly decreased at each reading in comparison with the values at week 0. There was a significant difference between YWF arm and YWF + gypsum arm at each reading

The levels of urine urate decreased in all three groups (*P* < 0.05). Analysis on FAS showed that there was a significant difference between the YWF group and the YWF + gypsum group at each reading (*P* < 0.05, Table 4, Fig. 3).

**Table 4:** Changes in urine urate (mean ± SD, mmol/24 h)

		Week 0	Week 1	Week 2	Week 3	Week 4
PPS	*YWF* arm	7.77 ± 0.39	7.59 ± 0.28*	7.42 ± 0.32*	7.33 ± 0.32*	7.33 ± 0.35*
	YWF + gypsum arm	7.82 ± 0.28	7.50 ± 0.33*	7.28 ± 0.44*	7.19 ± 0.45*	7.17 ± 0.48*
	Allopurinol arm	7.80 ± 0.31	7.53 ± 0.27*	7.32 ± 0.36*	7.24 ± 0.39*	7.21 ± 0.41*
FAS	*YWF* arm	7.80 ± 0.37	7.65 ± 0.31*	7.50 ± 0.36*	7.43 ± 0.38*	7.43 ± 0.41*
	YWF + gypsum arm	7.79 ± 0.33	7.48 ± 0.30*^†^	7.28 ± 0.40*^†^	7.21 ± 0.41*^†^	7.19 ± 0.44*^†^
	Allopurinol arm	7.78 ± 0.31	7.53 ± 0.26*	7.33 ± 0.35*	7.25 ± 0.36*	7.23 ± 0.40*

*In comparison with value at week 0, there was statistically significant difference (*P* < 0.05)

^†^In comparison with *YWF* arm at each reading, there was statistically significant difference (*P* < 0.05)

#### SF-36

The items of the SF-36 scale can be summarized as eight structures including physical functioning (PF), role-physical (RP), bodily pain (BP), general health (GH), vitality (VT), social functioning (SF), role-emotional (RE), and mental health (MH). All the changes in the eight structures of SF-36 during the treatment period were not significantly different between the three groups (data not shown).

#### CRP

In comparison with the values at week 0, the CRP level of the YWF significantly decreased at week 4 (*P* < 0.05) and there was no significant difference among the three groups at each weekend reading (Table 5).

**Table 5:** Changes in CRP (mean ± SD, mg/L)

		Week 0	Week 1	Week 2	Week 3	Week 4
PPS	*YWF* arm	14.43 ± 2.73	13.13 ± 1.82	13.34 ± 1.08	12.49 ± 0.64	10.09 ± 4.71*
	YWF + gypsum arm	13.66 ± 1.63	13.26 ± 1.81	12.98 ± 0.75	12.55 ± 0.85	11.56 ± 5.06
	Allopurinol arm	13.25 ± 1.32	12.99 ± 1.19	13.34 ± 1.15	12.56 ± 0.77*	11.03 ± 4.98
FAS	*YWF* arm	13.13 ± 2.63	12.87 ± 1.83	13.04 ± 1.30	12.33 ± 0.85	10.33 ± 4.34*
	YWF + gypsum arm	14.03 ± 3.40	13.03 ± 1.81	12.81 ± 0.96	12.45 ± 0.97	11.64 ± 4.62
	Allopurinol arm	13.15 ± 1.13	13.05 ± 1.31	13.20 ± 1.19	12.54 ± 0.76*	11.13 ± 4.77

*In comparison with value at week 0, there was a statistically significant difference (*P* < 0.05)

#### ESR

The ESR levels of the three arms fell in the scope of normal values and there was no significant change during treatment period (data not shown).

#### X-ray film

No individuals showed any significant changes on X-ray film digital image.

#### Acute flares

No acute flare was recorded in each group during treatment period.

### Adverse events

No adverse events were reported.

## Discussion

### Limitations

The lack of blinding of the present clinical study could result in performance bias and detection bias. Another limitation is that we were not sure whether our negative results resulted from the YWF had not been modified as an appropriate formula for patients with long gout history. We notice that the gout history in our study is, on average, 39–55 months. We consider whether the dampness and heat might have evolved as a “toxin.” The toxin accumulated in the blood and joints, resulting in hyperuricemia and gout arthritis. Modifying YWF by adding some TCM with property of detoxification may produce better clinical benefits of YWF.

### Generalizability

Our results of data analysis on FAS were largely consistent with those on PPS, which suggests that our results were sound. To our knowledge, in the field of TCM for gout, it is the first time that a clinical study was presented according to the CONSORT Extension for Chinese Herbal Medicine Formulas 2017 ([21], Additional file 1). Although the modification of *2WHP* may vary greatly due to the large number of TCM, this study is of clinical importance to those many TCM professionals who want to prescribe or receive the formulae modified, based on *2WHP*.

### Interpretation

A large amount of literature has investigated the conventional urate lowering medications for hyperuricemia in gout, such as febuxostat, allopurinol, and benzbromarone [22]. Febuxostat, given its established efficacy and safety, has been recommended as a suitable pharmacological option for first-line treatment of gout [23]. However, controversies remain: a systematic review and meta-analysis concluded that “There was no evidence that febuxostat is superior to allopurinol for clinically relevant outcomes. Given its higher cost, febuxostat should not be routinely used for chronic gout” [24]. Chen et al. [25] reported a traditional Chinese medicine prescription: Quzhuotongbi decoction could significantly lower the sUA levels in hyperuricemia model rats. Kodithuwakku et al. [26] reported that Shuang Qi gout capsules might be a potent anti-hyperuricemic agent. A systematic review concluded that Chinese herbal decoction and traditional western medicine led to similar clinical efficacy including lowering sUA [27].

A number of animal experiments reported that TCM showed hypouricemic effects [28, 29] and 2*WHP* series formulae [14, 16, 17] lowered the uric acid levels by diverse mechanisms. In a systematic review published in 2013, “25 out of 41 trials, involving 23 different herbal prescriptions, Li, et al., found statistical significance in lowering the serum uric acid level.” However, the authors admitted that the evidence was not decent due to “low quality of included trials” [30]. Generally speaking, in humans, the current literature reports TCM shows at least similar, if not better, clinical efficacy in comparison with western medicine [27]. We have carried out a systematic review where 19 RCTs and 1646 participants were included to evaluate the clinical efficacy of 2*WHP* series formulae and the results show that, compared with western medicine, the TCM formulae based on the 2*WHP* series could significantly decrease the level of sUA and increase the cure rate [31]. However, unexpectedly, the results of the present pilot RCT were not consistent with our previous systematic review findings: YWF failed to significantly decrease the sUA levels.

A large number of published clinical studies did not have the control arm [13, 15]. Many published RCTs claimed randomization, but none of them provide the details with respect to randomization. Due to the poor methodology involved in proper randomization, it appears that the current clinical evidence is not sound.

## Conclusions

The present clinical study, as a pilot trial, was exploratory rather than conclusive. We drew the following conclusions based on our research results: (1) YWM when modified on the basis of *2WHP* does not show significant hypouricemic effect; (2) Gypsum Fibrosum could provide additional effects to YWF in decreasing the urine urate levels but cannot add benefits to YWF in other outcome measures. Generally speaking, our clinical study is of optimal methodology and our conclusions were of desirable reliability.

## Additional file

Additional file 1: CONSORT Herbal checklist. (DOCX 15 kb)

## Abbreviations

2WHP: *Two Wonderful Herbs Powder*
3WHP: *Three Wonderful Herbs Pill*
4WHP: *Four Wonderful Herbs Pill*
ANOVA: Analysis of variance
BP: Bodily pain
CRP: C-reactive protein
ESR: Erythrocyte sedimentation rate
FAS: Full-analysis set
GCP: Good Clinical Practice
GH: General health
ITT: Intention-to-treat
mGLUT9: Mouse glucose transporter 9
MH: Mental health
mOAT1: Mouse organic anion transporter 1
mURAT1: Mouse urate transporter 1
PF: Physical functioning
PPS: Per-protocol set
RCT: Randomized controlled trial
RE: Role-emotional
RP: Role-physical
SF: Social functioning
sUA: Serum uric acid
TCM: Traditional Chinese Medicine
TKKD: *Three Kinds of Kernels Decoction*
VT: Vitality
XOD: Xanthine oxidase
YWF: *Yellow-dragon Wonderful-seed Formula*
