# Supplementary material for: “Yellow-dragon Wonderful-seed Formula” for hyperuricemia in gout patients with dampness-heat pouring downward pattern: a pilot randomized controlled trial
Source: Trials. 2018 Oct 11;19:551. doi: 10.1186/s13063-018-2917-8 (PMC6186073; doi:10.1186/s13063-018-2917-8)
Supplement: Supplementary file 1 — CONSORT Herbal checklist. (DOCX 15 kb) [file 13063_2018_2917_MOESM1_ESM.docx]

[**CONSORT Herbal**](http://www.equator-network.org/reporting-guidelines/consort-herbal/) **checklist**

| **Paper Section and Topic** | **Item Number** | **page number against each criterion** |
| --- | --- | --- |
| **Title and Abstract** | **1** | **P1-2** |
| **Introduction**  **Background** | **2** | **P3-5** |
| **Methods**  **Participants** | **3** | **P5** |
| **Interventions** | **4** | **P6-7** |
| **Objectives** | **5** | **P5** |
| **Outcomes** | **6** | **P7** |
| **Sample size** | **7** | **P7** |
| **Randomization**  **Sequence allocation** | **8** | **P7** |
| **Allocation concealment** | **9** | **P7** |
| **Implementation** | **10** | **P7** |
| **Blinding** | **11** | **P7** |
| **Statistical methods** | **12** | **P7-8** |
| **Results**  **Participant flow** | **13** | **P8, Figure 1** |
| **Recruitment** | **14** | **P8** |
| **Baseline data** | **15** | **P8, Table 2** |
| **Numbers analyzed** | **16** | **P8** |
| **Outcomes and estimation** | **17** | **P8-10, Figure 2-3, Table 3-5** |
| **Ancillary analyses** | **18** | **P8-10, Figure 2-3, Table 3-5** |
| **Adverse events** | **19** | **P10** |
| **Discussion**  **Interpretation** | **20** | **P11** |
| **Generalizability** | **21** | **P10** |
| **Overall evidence** | **22** | **P10-11** |
